# Interphotoreceptor Retinoid Binding Protein; Myths and Mysteries

**Published:** 2012-01

**Authors:** Federico Gonzalez-Fernandez

**Affiliations:** Medical Research Service, Veterans Affairs Medical Center, Buffalo, New York, USA; Departments of Ophthalmology, Ross Eye Institute, and Pathology & Anatomic Sciences; Graduate Program in Neurosciences State University of New York, and SUNY Eye Institute

Although it is the major soluble protein component of the extracellular matrix bathing the photoreceptors, little is known about the structure and function of interphotoreceptor retinoid-binding protein (IRBP). The critical anatomic compartment it occupies cannot be overemphasized as through the involution of the optic vesicle, the retinal pigmented epithelium (RPE) comes in contact with the neural retina.^1^ The zonula occludens and adherens of the RPE and external limiting membrane respectively restrict the interphotoreceptor matrix (IPM) to the “subretinal space”. Given its presence at this interface and early expression patterns, IRBP could have a function in retinal development,^2^,^3^ and recent studies point to a role in eye growth^4^. Early studies assumed that IRBP does not interact with components of the IPM or retina, as it is easily extracted from detached retinas by only gentle saline wash. This notion, recently revisited experimentally,^5^ has shown not to be completely true as saline wash leaves behind IRBP tightly bound to specific regions, particularly a domain surrounding the cone outer segments, presumably the cone matrix sheath (Fig. 1). An important question, which has received insufficient attention, is what are the binding partners that IRBP interacts with in the IPM and/or on the cell surface?

Its name is unfortunate, as it does not remind us that IRBP is present elsewhere besides the IPM. IRBP is expressed by the pineal gland in man.^6^,^7^ Its function there is completely unknown. Equally unknown is the role of IRBP in the vitreous, one of the locations where IRBP was initially described. Interestingly, recent studies have shown decreased levels of IRBP in the vitreous in the early stages of diabetes.^8^ The source and turnover of vitreous IRBP is unknown although its mRNA has been detected in the ciliary epithelium.^9^ Finally, in the retina, IRBP is not restricted to the IPM as was commonly assumed, but is internalized avidly by the RPE (see below). Certainly, there will be much to be learned about IRBP as studies examine its function beyond the IPM.

IRBP is secreted into the IPM by the rods and cones, and interestingly by the RPE in some cases.^10^ The exclusion limit of the *zonula adherens* restricts it to the subretinal compartment. Despite restriction to the subretinal compartment, IRBP is rapidly turned over probably by RPE and photoreceptor endocytosis.^11^,^12^ The function of this interesting turnover is unknown, but may be linked to circadian changes in mRNA expression as part of a complex mechanism regulating IPM IRBP concentrations.^13^ A potential receptor mediated photoreceptor uptake mechanism needs to be revisited.^14^ IRBP turnover may be related to its function in transporting hydrophobic molecules, part of a degradation pathway, or both. These important and interesting questions remain largely unexplored.

The importance of IRBP to the retina has recently been underscored by its role in disease. Early studies noted reduced quantities of IRBP in the rod-cone degeneration in Abyssinian cats. Photoreceptor degeneration occurs in transgenic mice lacking IRBP. A mutation in IRBP has been associated with a form of autosomal recessive retinitis pigmentosa^15^ (Fig. 2B). Although the mechanism leading to the degeneration is largely unknown, these studies indicate that the presence of sufficient quantities of functional IRBP is critical to photoreceptor survival. Finally, IRBP is one of the most useful antigens in experimental autoimmune uveitis, and has been implicated in spontaneously occurring uveitis in animals and man.

IRBP was discovered three decades ago and shown to carry endogenous all-*trans*, 11-*cis* retinol and 11-*cis* retinal in a light dependent manner. However, its role in the visual cycle is still far from clear. The early prevailing view that IRBP simply solubilizes retinoids in the aqueous IPM milieu has never been completely satisfying, and not always born out experimentally as an absolute requirement for retinoid trafficking. At the same time, the true complexity of the visual cycle has become more apparent as briefly summarized in Fig. 2A. The remarkable complexity of the retinoid trafficking taking place across the IPM is certainly greater than was anticipated at the time of initial descriptions of IRBP. Following its photoisomerization, this trafficking begins with the release of all-*trans* retinol into the IPM. From there, it may enter the RPE or Muller cells to be enzymatically re-isomerized to the 11-*cis* isomer, which is returned through the IPM to the outer segments. A difference between the rod and cone visual cycles is that the RPE provides 11-*cis* retinal, while Muller cells release 11-*cis* retinol. It is thought that in this way, the cone visual cycle ensures a protected supply of the 11-*cis* isomer to the cones which are endowed with an 11-*cis* retinol dehydrogenase, allowing utilization of 11-*cis* retinol for pigment regeneration.^16^

How might IRBP function in this complex cycle? The visual cycle must accomplish the efficient delivery of three different retinoids (11-*cis*, all-*trans* retinol and 11-*cis* retinal) to four different cell types (rod, cone, RPE and Muller) at the correct time, protecting retinoids from isomeric and oxidative degradation. These tasks must be accomplished under large extremes of retinoid flux encountered in night and daytime vision. One possibility is that IRBP contains ligand-binding sites tailored for each visual cycle retinoid, and docking sites that can distinguish cell surface and IPM binding sites.^17^,^18^ Mechanisms must exist to regulate ligand affinities to accomplish the appropriate retinoid uptake or release. Some of the pieces of the puzzle are emerging.^19^–^21^ IRBP is known to promote outer segment release of all-*trans* retinol,^22^ and its delivery to the RPE. IRBP also enhances both the release of 11-*cis* retinal from the RPE, and its return to the outer segments. However, the molecular mechanism behind these functions remains unknown. Recent studies suggest an important role of IRBP in the cone visual cycle.^23^,^24^ Finally, a role of IRBP in protecting the oxidative and isomeric state of visual cycle retinoids^24^,^25^ is an exciting possibility that may reflect a thiol dependent antioxidant activity of the protein^26^. Uncovering the mechanisms of IRBP functions will require further detailed information on its structure.

IRBP is certainly unique among retinoid-binding proteins as it is composed of two to four homologous “modules”, each ∼300 amino acids in length.^27^ The significance of this structure, which makes IRBP the largest known retinoid-binding protein, is largely unknown. The individual modules, which are homologous with carboxyl-terminal processing proteases (CPTases) and crotonases, represent functional units of the protein. Photosystem II D1 CPTase (D1P), a prototypical CPTase, plays a role in the repair of oxidative damage to the reaction center by renewing the photosystem II D1P. The repair involves replacement of damaged D1P with new protein. During this replacement, D1P catalyzes the hydrophobic C-terminal cleavage of the new D1P. Each module of IRBP is structurally similar to the D1P domains A and C which correspond to the N- and C-terminal IRBP modules, respectively. Furthermore, the C-terminal domain B of the IRBP modules and domain C of D1P exhibit a structural homology with crotonases (enoyl Co-A hydratase/isomerases). The X-ray structure of the second module of *Xenopus* module 2 shares with these enzymes a structural core composed of three helices, and a five-stranded β-sheet in domain B forming a ββα-spiral fold.^28^ In view of IRBP’s homology with these two enzyme families, an enzymatic activity for IRBP should be kept in mind. The vitamin A binding domain of IRBP lies in a separate hydrophobic cavity. The shallow cleft formed by the fold was assumed to represent the retinol-binding site. However, a second hydrophobic site consisting of a highly restricted cavity was more recently appreciated during *in silico* ligand-docking studies. Site directed mutagenesis studies indicate that this hydrophobic site represents the retinoid-binding site^29^ (Fig. 2B).

Initially viewed as only providing a hydrophobic ligand-binding protein for retinoids crossing the IPM, IRBP appears to mediate a variety of specific roles particularly targeting the cellular delivery and release of visual cycle retinoids while protecting these molecules from isomeric and oxidative degradation. The complexity of the structure of IRBP and the visual cycles was not anticipated when IRBP was first described three decades ago. Emerging molecular and structural data taken together with increased understanding of the visual cycle, and ocular development promise to elucidate the function of this interesting protein. It is critical that potential roles for IRBP outside of the visual cycle and retina not be ignored, but addressed experimentally. Clearly, there is much more to be discovered compared to what has already been uncovered regarding the function of this protein and its role in disease.

## Figures and Tables

**Figure 1.**
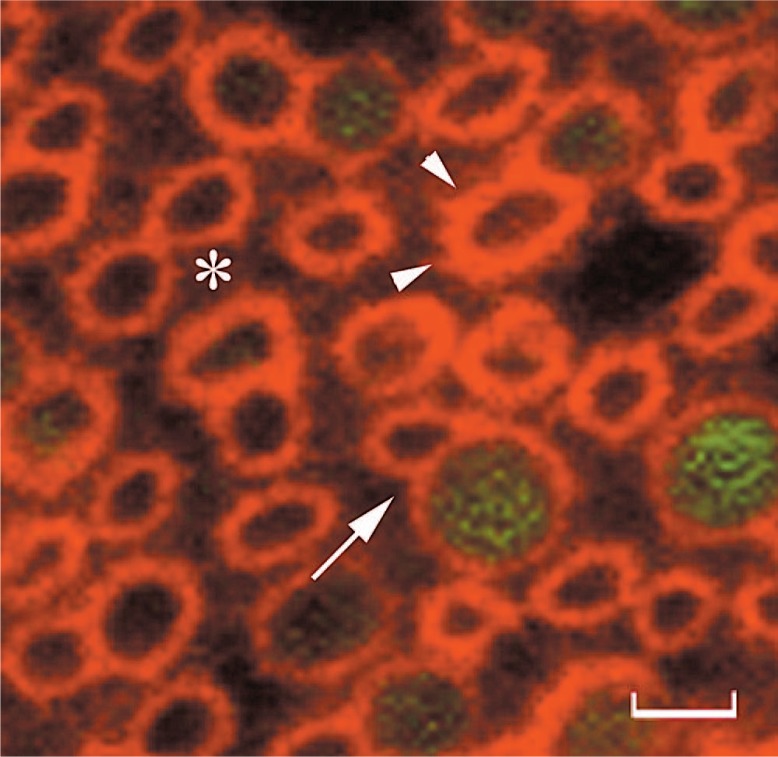
IRBP interacts with the cone matrix sheath. This photomicrograph of chicken cones in cross-sectional orientation shows the distribution of IRBP by indirect immunofluorescence following saline wash. The retinas were washed three times, flat mounted, and probed with mAb F7 anti-IRBP, followed by goat anti-mouse IgG-647. The saline wash removed IRBP from the regions between cone matrix sheaths (asterisk) leaving behind that bound to a matrix domain rimming the outer segments (arrow heads). Represented here are merged confocal fluorescence images at 633 nm (red, IRBP) and 488 nm (green, oil droplet autofluorescence). Arrow, double cone; scale bar = 3.3 μm. Adapted from Garlip et al. J. Comp. Neurol. (in press) with permission from Wiley-liss, Inc.

**Figure 2.**
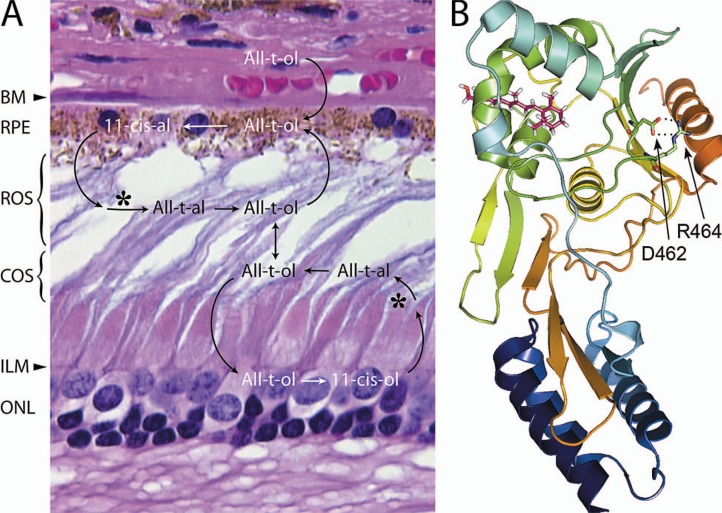
Overview of the visual cycle, and structure of an IRBP module. A) The diagram is overlaid on a hematoxylin & eosin stained paraffin section of human retina (near the fovea). Interphotoreceptor matrix (IPM) glyconjugates are largely responsible for the basophilic outer segment staining. The apparent spaces among the outer segments is a histological processing artifact. The larger cone nuclei are located near the external limiting membrane (ELM, arrowhead). Bruch’s membrane (BM, arrow head); outer nuclear layer (ONL). 11-cis retinal (11-cis-al) is photoisomerized (*) to its 11-cis isomer, which is reduced to all-trans retinol (All-t-ol). The All-t-ol is then released from rod and cone outer segments (ROS and COS respectively) into the IPM. B) Ribbon representation of the module II Xenopus IRBP structure docked with all-t-ol (magenta).The N- and C-terminal regions are shown in blue and red respectively. A salt bridge (dotted lines) extends between the carboxamide side group of D462, and guanidinium group of R464. These highly conserved residues are shown in stick representation (oxygen, red; nitrogen, blue). The corresponding aspartic acid of human IRBP is replaced by asparagine in a form of autosomal recessive retinitis pigmentosa. It is possible that this substitution (D1080N), by abolishing the conserved salt bridge, destabilizes the nearby retinoid-binding site. The structure shown in this panel is adapted from Hollander et al. Invest. Ophthal. Vis. Sci. 50:1864–72, 2009 with permission from the Association for Research in Vision and Ophthalmology.
